# Hypotension artérielle per-anesthésique du sujet âgé lors d’une chirurgie urgente: quels facteurs de risque?

**DOI:** 10.11604/pamj.2017.26.242.9886

**Published:** 2017-04-27

**Authors:** El Hadji Boubacar Ba, Papa Alassane Leye, Mamadou Mour Traoré, Pape Ibrahima Ndiaye, Ibrahima Gaye, Mamadou Diawo Bah, Mamadou Lamine Fall, Elisabeth Diouf

**Affiliations:** 1 Service d’Anesthésie-Réanimation CHU le Dantec, Faculté de Médecine UCAD, Dakar, Sénégal; 2 Service d’Anesthésie-Réanimation HEAR de Fann, Faculté de Médecine UCAD, Dakar, Sénégal

**Keywords:** Anesthésie, hypotension artérielle, sujet âgé, Anesthesia, hypotension, elderly patient

## Abstract

L'anesthésie des personnes âgées de 65 ans et plus en urgence reste complexe. La survenue d'incidents peropératoires et en l'occurrence l'hypotension artérielle est conditionnée par leur état de santé initial et par la qualité de la prise en charge péri-opératoire. L'étude a pour but de déterminer l'incidence de l'hypotension artérielle per-anesthésique du sujet âgé pour une chirurgie urgente et évaluer l'implication de certains facteurs dans sa survenue: âge, sexe, terrain, classe ASA, technique anesthésique. Une étude rétrospective descriptive et analytique a été réalisée aux blocs des urgences chirurgicales du CHU Aristide LE DANTEC allant du 1er mars 2014 au 28 février 2015. Nous avons colligé 210 patients sur 224 anesthésies en urgence du sujet âgé de 65 ans et plus, soit 10,93%. On notait 101 hommes et 109 femmes dont les 64,3% présentaient au moins une tare. L'évaluation de l'état préopératoire des patients a été faite avec la classification de l'American Society of Anesthesiology (ASA), avec 71% pour les classes ASA 1 et 2 et 29% pour les classes ASA 3 et 4. L'anesthésie locorégionale était la technique anesthésique la plus pratiquée (56,7%). L'hypotension artérielle peropératoire a été objectivée chez 28 patients soit 13,33%, dont 16 cas sous anesthésie générale et 12 cas sous anesthésie locorégionale. Elle était plus fréquente chez les patients de classe ASA élevée et un peu moins sur terrain d'HTA et de cardiopathie sous-jacente. L'anesthésie du sujet âgé en urgence expose à un risque d'hypotension artérielle peropératoire non négligeable notamment chez les patients de classes ASA élevée. Sa prévention repose sur une bonne évaluation préopératoire et une prise en charge anesthésique adéquate.

## Abstract (en)

Emergency anesthesia in elderly patients aged 65 years and older is complex. The occurrence of intraoperative incidents and arterial hypotension is conditioned by patients' initial health status and by the quality of intraoperative management. This study aimed to determine the incidence of intra-anesthetic arterial hypotension in elderly patients during emergency surgery and to assess the involvement of certain factors in its occurrence: age, sex, patient's history, ASA class, anesthetic technique. We conducted a retrospective descriptive and analytical study in the Emergency Surgery Department at the Aristide Le Dantec University Hospital from 1 March 2014 to 28 February 2015. We collected data from 210 patients out of 224 elderly patients aged 65 years and older undergoing emergency anesthesias (10.93%). Data of 101 men and 109 women were included in the analysis, of whom 64.3% had at least one defect. Patients' preoperative status was assessed using American Society of Anesthesiology (ASA) classification: 71% of patients were ASA class 1 and 2 and 29% were ASA class 3 and 4. Locoregional anesthesia was the most practiced anesthetic technique (56.7%). 28 patients (13.33%) had intra-anesthetic arterial hypotension, of whom 16 under general anesthesia and 12 under locoregional anesthesia. It was more frequent in patients with high ASA class and a little less frequent in patients with PAH and underlying heart disease. Arterial hypotension in elderly patients during emergency surgery exposes the subject to the risk of not negligible intraoperative hypotension, especially in patients with high ASA class. Prevention is based on adequate preoperative assessment and anesthetic management.

## Introduction

L'anesthésie des personnes âgées de 65 ans et plus en urgence reste complexe. La survenue d'incidents peropératoires et en l'occurrence l'hypotension artérielle est conditionnée par leur état de santé initial et par la qualité de la prise en charge péri-opératoire. Le maintien de la pression artérielle tout au long de la période opératoire est un des impératifs de la prise en charge péri opératoire, en particulier chez les sujets à risque cardiovasculaire [1]. Le but de notre étude était de déterminer l'incidence de l'hypotension artérielle per-anesthésique du sujet âgé pour une chirurgie urgente et d'évaluer l'implication de certains facteurs dans sa survenue notamment l'âge, le sexe, le terrain, la classe ASA, et la technique anesthésique.

## Méthodes

Il s'agissait d'une étude rétrospective, descriptive et analytique portant sur tous les patients âgés de 65 ans ou plus et ayant subi une intervention chirurgicale aux blocs opératoires des urgences chirurgicales du CHU Le DANTEC. Cette étude s'étendait sur une période de 12 mois, allant du 1^er^ mars 2014 au 28 février 2015. Les patients ont été colligés à partir du registre d'anesthésie des urgences chirurgicales et des fiches d'anesthésie. Sur ces fiches étaient notées les données de l'évaluation préopératoire, de la prise en charge anesthésique et chirurgicale, ainsi que les incidents et /ou accidents peropératoires. Tous les patients avaient bénéficié d'une évaluation préopératoire comportant un interrogatoire et un examen clinique. La prescription des examens complémentaires n'était pas systématique, elle était faite en fonction du terrain, de la pathologie chirurgicale, des données cliniques, de la disponibilité de ces examens. Les éléments recueillis ont été: l'âge, le sexe, le terrain, la classe ASA, la technique anesthésique, la survenue d'une hypotension artérielle peropératoire. L'hypotension artérielle était définie par une pression artérielle systolique < 90 mmHg. Les données ont été analysées sur le logiciel Sphinx plus et le seuil de significativité était fixé à p < 0,05.

## Résultats

Sur une période de douze mois, 224 patients de 65 ans et plus ont été recensés sur 2049 patients opérés aux blocs des urgences pendant la période d'étude soit 10,93%. Cependant, 14 fiches d'anesthésie n'ont pas été retrouvées, réduisant notre série à 210 patients. Cinquante-deux pourcent des patients étaient de sexe féminin. L'âge moyen était de 74,9 ans [65-96 ans] avec une prédominance des gérontins [65-74 ans]. La majorité (64,3%) des patients avait un terrain particulier et les tares les plus fréquentes étaient : le diabète (41,9%), l'HTA (37,1%), la cardiopathie (4,76%). L'évaluation préopératoire des patients a révélé que les classes ASA 1 et ASA 2 représentaient 71%, 23% pour la classe ASA 3 et 6% la classe ASA 4. L'anesthésie locorégionale (ALR) était la technique anesthésique la plus pratiquée 119 cas soit 56,7% avec 32,5% d'ALR périphérique, 14,76% de rachianesthésie (RA) continue, 8,09% de RA conventionnelle et deux cas de RA unilatérale. Vingt-huit cas d'hypotension artérielle ont été notées soit 13,33%. Le sexe (Tableau 1) n'avait pas d'effet sur la survenue d'hypotension artérielle p = 0,8496. On notait une légère augmentation de l'incidence de l'hypotension avec l'âge (Tableau 1), mais la corrélation statistique n'était pas significative p à 0,8634. L'incidence de l'hypotension artérielle sur terrain d'HTA était plus marquée (Tableau 1), même si l'existence de tare ne semble pas avoir un rôle déterminant dans la survenue d'hypotension artérielle (p=0,0901). Le lien entre la classe ASA et la survenue d'hypotension artérielle (Tableau 2) était important et ceci d'autant plus que la classe était élevée : p= 0,0454. La RA conventionnelle et l'anesthésie générale étaient associées à plus d'hypotension per opératoire (Tableau 3), cependant l'implication de la technique anesthésique sur la survenue d'hypotension artérielle per opératoire n'était que peu significative p à 0,1132.

**Tableau 1:** Rôle des facteurs démographiques et du terrain dans la survenue d’hypotension artérielle

		Nombre de patients (n)	Hypotension (n)	Pourcentage (%)
**Age** p=0,8634	65-74 ans	123	16	13,00
	75-84 ans	63	9	13,6
	85 ans et plus	21	3	14,23
**Sexe** p=0,8496	Féminin	109	12	11,00
	Masculin	101	16	15,84
**Terrain** p=0,0901	HTA	78	12	15,38
	Diabète	88	7	7,90
	Cardiopathie	20	2	10,00
	Pas de tare	75	7	9,30

**Tableau 2:** Rôle de la classe ASA dans la survenue d’hypotension artérielle

Classe ASA	Nombre de patients (n)	Hypotension (n)	Pourcentage (%)
ASA 1	24	3	12,5
ASA 2	124	13	10,48
ASA 3	49	7	14,24
ASA 4	13	5	38,46

Dépendance statistique significative p = 0,0454

**Tableau 3:** Rôle de la technique anesthésique dans la survenue d’hypotension artérielle

Technique anesthésique	Nombre de patients (n)	Hypotension (n)	Pourcentage (%)
AG	91	16	17,58
RA conventionnelle	17	6	35,29
RA continue	31	3	9,67
RA unilatérale	2	0	0
ALR périphérique	69	3	4,34

Dépendance statistique peu significative p = 0,1132

## Discussion

L'hypotension induite par l'anesthésie résulte à la fois d'un effet direct des agents d'anesthésie sur le myocarde et/ou les vaisseaux périphériques et d'un effet indirect secondaire à l'effet de l'anesthésie sur le système nerveux qui diminue le tonus des systèmes neurohumoraux en particulier le système sympathique [1]. Son traitement symptomatique fait appel au remplissage vasculaire et à l'administration de vasopresseurs [2].

Elle a été reconnue comme la plus fréquente des complications per opératoires au cours de l'anesthésie du sujet âgé. Dembélé M. G. [3] avait retrouvé une incidence de 49%, Moore et Bridenbaugh [4] rapportent 38,5 % d'hypotension artérielle liée à l'anesthésie locorégionale. Ailleurs, globalement l'incidence de l'hypotension artérielle au cours de la rachianesthésie varie entre 15 et plus de 50% selon la littérature [5, 6]. Dans notre étude, elle était survenue à des périodes différentes au cours de l'intervention et a été surtout observée chez les patients ayant bénéficié d'une AG (17,58%) et d'une RA conventionnelle (35,29%). Elle était moins fréquente sous ALR périphérique (4,34%), RA titrée (9,69%), et elle était nulle dans les deux cas de RA unilatérale.Il a été noté dans les travaux de Minville et coll comparant la rachianesthésie conventionnelle et la rachianesthésie continue chez des personnes âgées opérées pour une fracture du col du fémur que la fréquence des épisodes d'hypotension artérielle était significativement plus importante dans le groupe de la rachianesthésie standard avec 68% [7]. Ainsi la RA continue constituerait une technique intéressante dans la diminution de la fréquence des hypotensions per-anesthésiques comme l'a montré l'étude de Beye et al [8] qui retrouvait une incidence de 6,7%. Il ressort de notre étude que la survenue d'hypotension artérielle en per opératoire était indépendante de l'âge (p = 0,8634), et du sexe (p = 0,8496). Le lien n'était que peu significatif avec le terrain de nos patients (p = 0,0901) et la technique anesthésique pratiquée (p = 0,1132). Cependant, la dépendance à la classe ASA était importante et ceci d'autant plus que la classe ASA était élevée avec un P value à 0,0454. Par ailleurs, peu d'étude ont identifié de manière fiable les facteurs favorisants la survenue d'hypotension artérielle per opératoire, il s'agit de: l'âge avancé, la persistance de facteurs de co-morbidité et la grossesse [9, 10].

## Conclusion

L'anesthésie du sujet âgé en urgence expose à un risque d'hypotension artérielle peropératoire non négligeable. Notre étude n'a objectivé que la classe ASA élevée comme facteur déterminant de l'hypotension artérielle per-anesthésique chez le sujet âgé. Sa prévention repose sur une bonne évaluation préopératoire et une prise en charge anesthésique adéquate.

### Etat des connaissances actuelle sur le sujet

- L'hypotension artérielle, est la plus fréquente des complications per-anesthésiques chez le sujet âgé;
- Elle est déterminée par certains facteurs dont l'âge, les facteurs de comorbidité;
- Elle est moins fréquente en cas d'anesthésie locorégionale.

### Contribution de notre étude à la connaissance

- L'âge ne constitue pas un facteur déterminant de la survenue d'hypotension artérielle per anesthésique chez le sujet âgé, c'est surtout l'état préopératoire du patient notamment la classe ASA.
